# Machine learning algorithms and their predictive accuracy for suicide and self-harm: Systematic review and meta-analysis

**DOI:** 10.1371/journal.pmed.1004581

**Published:** 2025-09-11

**Authors:** Matthew J. Spittal, Xianglin Aneta Guo, Laurant Kang, Olivia J. Kirtley, Angela Clapperton, Keith Hawton, Nav Kapur, Jane Pirkis, Greg Carter

**Affiliations:** 1 Centre for Mental Health and Community Wellbeing, Melbourne School of Population and Global Health, The University of Melbourne, Melbourne, Australia; 2 Hunter New England Local Health District, Waratah, Australia; 3 Center for Contextual Psychiatry, KU Leuven, Leuven, Belgium; 4 Centre for Suicide Research, Department of Psychiatry, University of Oxford, Warneford Hospital, Oxford, United Kingdom; 5 National Confidential Inquiry into Suicide and Safety in Mental Health (NCISH), Centre for Mental Health and Safety, Faculty of Biology, Medicine and Health, University of Manchester, Manchester, United Kingdom; 6 NIHR Greater Manchester Patient Safety Research Collaboration, University of Manchester, Manchester, United Kingdom; 7 Mersey Care NHS Foundation Trust, Liverpool, United Kingdom; 8 College of Health, Medicine and Wellbeing, School of Medicine and Public Health, The University of Newcastle, Callaghan, Australia; 9 Department of Consultation Liaison Psychiatry, Calvary Mater Newcastle Hospital, Waratah, Australia; Massachusetts General Hospital, UNITED STATES OF AMERICA

## Abstract

**Background:**

There has been rapid expansion in the development of machine learning algorithms to predict suicidal behaviours. To test the accuracy of these algorithms for predicting suicide and hospital-treated self-harm, we undertook a systematic review and meta-analysis. The study was registered (PROSPERO CRD42024523074).

**Methods and findings:**

We searched PubMed, PsycINFO, Scopus, EMBASE, IEEE, Medline, CINALH and Web of Science from database inception until 30 April 2025 to identify studies using machine learning algorithms to predict suicide, self-harm and a combined suicide/self-harm outcome. Studies were included if they examined suicide or hospital-treated self-harm outcomes using a case-control, case-cohort or cohort study design. Studies were excluded if they used self-reported outcomes or examined outcomes using other study designs. Accuracy was assessed using statistical methods appropriate for diagnostic accuracy studies. Fifty-three studies met the inclusion criteria. The area under the receiver operating characteristic curves ranged from 0.69 to 0.93. Sensitivity was 45%–82% and specificity was 91%–95%. Positive likelihood ratios were 6.5–9.9 and negative likelihood values were 0.2–0.6. Using in-sample prevalence values, the positive predictive values ranged from 6% to 17%. Using out-of-sample prevalence values at an LR+ value of 10, the positive predictive value was 0.1% in low prevalence populations, 17% in medium prevalence populations and 66% in high prevalence populations. The main study limitations were the exclusion of relevant studies where we could not extract sufficient information to calculate accuracy statistics and between-study differences in the follow-up time over which the outcomes were observed.

**Conclusions:**

The accuracy of machine learning algorithms for predicting suicidal behaviour is too low to be useful for screening (case finding) or for prioritising high-risk individuals for interventions (treatment allocation). For hospital-treated self-harm populations, management should instead include three components for all patients: a needs-based assessment and response, identification of modifiable risk factors with treatment intended to reduce those exposures, and implementation of demonstrated effective aftercare interventions.

## Author summary

Why was this study done?

## Abstract 3

In a systematic review, Matthew Spittal and colleagues investigate the accuracy of machine learning algorithms to predict suicide and self-harm. They find the predictive properties of these machine learning algorithms to be poor, and no better than traditional risk assessment scales.

- Numerous risk assessment scales have been developed over the past 50 years to identify patients at high risk of suicide or self-harm. These scales classify patients as either at high or low risk, and treatment pathways are frequently based on the results of this assessment.
- In general, these scales have poor predictive accuracy, and this is one of the reasons why many clinical practice guidelines strongly discourage risk assessment for suicide and self-harm.
- The availability of modern machine learning methods and access to electronic health record and registry data has re-focussed attention on developing new algorithms to predict suicide and self-harm.

## What did the researchers do and find?

- We undertook a systematic review and meta-analysis to summarise the predictive properties of machine learning algorithms to predict suicide and self-harm.
- The overall quality of the research in this area was poor, with most studies at either high or unclear risk of bias.
- We found that the predictive properties of these machine learning algorithms were poor and no better than traditional risk assessment scales.

## What do these findings mean?

- Machine learning algorithms incorrectly classify more than half the people who subsequently present to hospital for self-harm or die by suicide as low risk.
- A classification of high risk poorly forecasts who will engage in suicide or self-harm.
- There is insufficient evidence to warrant changing recommendations in current clinical practice guidelines about risk assessment.
- The findings are limited by the exclusion of studies where we could not extract the information required to undertake a meta-analysis and by the included studies assessing the outcomes over different time periods.

## Introduction

Numerous studies have sought to identify patients at high risk of suicide or self-harm so that treatment can be provided specifically to them [1,2]. The risk assessment scales that have been developed stratify patients into high or low risk categories, with treatment pathways based on the classification [3]. The main clinical group that has been the focus of risk stratification is patients treated for self-harm (self-poisoning or self-injury) in the general hospital setting. Patients classified as high risk are typically prioritised for more intensive aftercare interventions than patients classified as low risk. Immediate interventions are classically psychiatric inpatient admission, close nurse observation or more urgent, frequent or intense community-based treatment (supervision). A high-risk classification, however, is not necessary to allocate effective, longer-term therapy-based interventions for suicidal behaviours like cognitive behavioural therapy in unselected self-harm populations [4], dialectical behaviour therapy in selected populations [5] or for suicide prevention in various clinical populations [6].

There is clear evidence that the traditional risk assessment scales used to predict suicide or self-harm have modest sensitivity and low positive predictive values [7–9]. In keeping with these findings, clinical guidelines do not recommend using risk stratification to allocate treatment in hospital-treated self-harm populations, and the US Preventive Services Task Force does not recommend screening for suicide risk in primary care [10–12], although conversely, the US Joint Commission recommends screening for suicide ideation for all patients over 12 years of age in all behavioural health services [13].

Efforts to improve risk prediction have recently focussed on using machine learning to develop algorithms that can predict suicide and self-harm. Machine learning is a branch of artificial intelligence in which prediction algorithms are developed by automatically and iteratively testing for complex associations between many factors in a dataset. Many studies emphasise the improved accuracy of their algorithms [14], suggesting that the poor accuracy of the traditional instruments has been overcome. But an important limitation of some of these studies, is a reliance on a case-control data to develop and evaluate algorithms. The use of the case-control design in diagnostic accuracy studies has previously been criticised as this design overestimates accuracy [15]. This overestimate occurs because the prevalence of the outcome is determined by the study design, and it is common in case-control studies to use a sample comprising half cases and half controls (meaning the apparent prevalence is 50%). The positive and negative predictive values of any risk score, however, are closely related to the prevalence of the outcome [16]. Suicide and self-harm are rare events, even in populations where the prevelence of these behaviours is high [14]. Thus, the high positive predictive values reported in some studies may be an artefact of the case-control design. This criticism is less likely to apply to cohort studies, although the retrospective nature of many cohort studies, where exposure data are collected when the outcome is already known, may be another potential source of bias.

To test the predictive accuracy of risk prediction algorithms developed using machine learning techniques, we undertook a systematic review and meta-analysis, paying particular attention to study design issues and their implications for prevalence. Our goal was to estimate a range of accuracy statistics of algorithm performance, namely, the area under the curve, sensitivity, specificity, likelihood ratios and positive and negative predictive values. We focused on studies that predict either hospital-treated self-harm or suicide mortality as these are clinically relevant outcomes used by clinicians to differentially allocate treatment for high-risk patients and which usually rely on the same institutional data sources to identify outcomes for all participants.

## Methods

We report our study using the Preferred Reporting Items for Systematic Reviews and Meta-Analyses (PRISMA 2020) statement [17] (S1 PRISMA Checklist). The study was registered (PROSPERO: CRD42024523074). Screening, full text review, data extraction and quality assessment were undertaken using Covidence.

### Search strategy and selection criteria

We searched PubMed, PsycINFO, Scopus, EMBASE, IEEE, Medline, CINALH and Web of Science from database inception until 30 April 2025 with the following search terms (“suicid*” OR “self?harm”) AND (“risk” OR “predict*” OR “class*”) AND (“machine learning”). No language restrictions were applied. We screened reviews, editorials and commentaries for further references. Titles and abstracts were screened independently by two authors. These studies were then assessed for eligibility in full-text review by the same authors. Disagreements were resolved by consensus.

Studies were eligible for inclusion if (a) the outcome was suicide or hospital-treated self-harm or a composite of these two; (b) the study involved primary research using a case-control, case-cohort or cohort design; (c) the study reported on a machine learning algorithm resulting in two or more risk factors measured at the individual level; (d) the study reported outcomes for any population or subgroup within the population (e.g., psychiatric treatment populations, people treated for self-harm); and (e) the study reported sufficient data to extract the number of true positives, false positives, false negatives and true negatives.

We excluded studies if (a) they only used suicidal ideation as the outcome; (b) used self-reported outcomes (e.g., self-reported suicide attempt or self-reported suicide risk); (c) the outcome was a specific suicide method (e.g., suicide by firearm); or (d) they only used aggregate predictors such as the number of firearm stores in an area.

### Data extraction

The following data were extracted for each study: the lead author and publication year, title, country where the study was conducted, study population, study design (case-control, case-cohort, cohort), data source, study outcomes (suicide, self-harm, or a combined suicide/self-harm endpoint), machine learning method, time frame over which the outcome was assessed (30 days, 60 days, 90 days, 180 days, 1 year, other), and for each outcome, the number of true positives, false positives, false negatives and true negatives. If multiple thresholds were reported, diagnostic values were extracted at the 95th percentile as this is a commonly used threshold in this literature. If diagnostic values were reported at multiple time points, the longest time point was selected as this will give the most optimistic positive predictive value. Where possible, results from validation samples were extracted. For studies reporting multiple diagnostic values from different algorithms, we prioritised extracting results for the best-fitting model as identified by the authors. If this was unclear, results from the algorithm with the highest sensitivity was instead prioritised. Where results were stratified by sex, these were combined into an overall count. If data from different cohorts were reported, we extracted results from mental health service cohorts. Data were independently extracted by two authors with disagreements resolved by consensus.

### Quality and risk of bias

The quality of each study was assessed in two ways. We examined if there was an explicit statement that the study reported against a relevant guideline (e.g., Transparent Reporting of a Multivariable Prediction Model for Individual Prognosis or Diagnosis (TRIPOD) [18] or Standards for Reporting of Diagnostic Accuracy Studies (STARD) [19]). We then examined adherence to the TRIPOD checklist. Risk of bias was assessed using the second revision of the Quality Assessment of Diagnostic Accuracy Studies instrument (QUADAS 2) [20]. Assessment was done by two authors with disagreements resolved by a third author.

### Statistical analyses

We conducted our meta-analyses in four stages. First, we estimated the pooled area under the receiver operating characteristic curve (AUROC) [21]. Next, we estimated the pooled sensitivity and specificity using a bivariate random effects meta-analysis [22]. This method, also known as a Reitsma model, jointly estimates the pooled sensitivity and specificity after a logit transformation by also estimating the negative correlation between these two estimates. We estimated heterogeneity at this stage using the adjusted *I*^2^ statistic [23] and plotted the summary receiver operating characteristic curves (sROC). The adjusted *I*^2^ statistic was developed for the meta-analyses of diagnostic accuracy studies and adjusts for sample size, the largest source of heterogeneity in these types of studies. The sROC is similar to a forest plot except that it plots study-specific estimates on two dimensions, *sensitivity* and the *false positive rate* (i.e., 1 − specificity). Third, we estimated the pooled positive and negative likelihood ratios (LR+ and LR−) using the method proposed by Zwinderman and Bossuyt [24]. They recommend estimating these measures by sampling the sensitivities and specificities derived from the analysis described above using the bivariate normal distribution and then calculating the LR values in each sample. We therefore drew 100,000 samples using a Monte Carlo Markov chain. For each sample, we calculated LR+ and the LR− and then estimated the sample mean and 95% credible intervals (the 2.5 and 97.5 percentiles of the samples). Fourth, we used Bayes’ rule to estimate the in-sample positive and negative predictive values. Under Bayes’ rule, the positive and negative predictive values are a function of the baseline prevalence of the outcome and the likelihood ratios [25]. We estimated the baseline prevalence from the cohort studies and applied these to both cohort and case-control likelihood ratios. Baseline prevalence was calculated using a random effects meta-analysis, where the proportions were transformed using the standard arcsine transformation prior to analysis. The back-transformed prevalences were therefore used as the pre-test probabilities. We report the median positive and negative predictive values and their 2.5 and 97.5 percentiles. All these analyses were stratified by outcome (suicide, self-harm, suicide/self-harm) and study design (case-control, cohort). Case-cohort and cohort studies were grouped together because case-cohort studies are a subset of cohort studies. We only undertook meta-analysis when data from five or more studies were available for analysis.

To examine how a hypothetical algorithm with indicative accuracy would perform in different clinical populations, we estimated out-of-sample positive predictive values using 1-year baseline prevalences from six different populations for varying LR+ values. These populations (and outcomes) were suicide in general population (0.01%), suicide after discharge from inpatient psychiatric facility (0.5%), self-harm in general population (1.5%), suicide after discharge for self-harm (2.0%), self-harm after discharge from inpatient psychiatric facility (6.5%) and self-harm after discharge for self-harm (16%). These prevalence estimates were drawn from the literature [26–30].

All analyses were undertaken in R version 4.4.2, with the meta-analyses undertaken using the mada and metafor packages [21,31].

## Results

**Fig 1:**
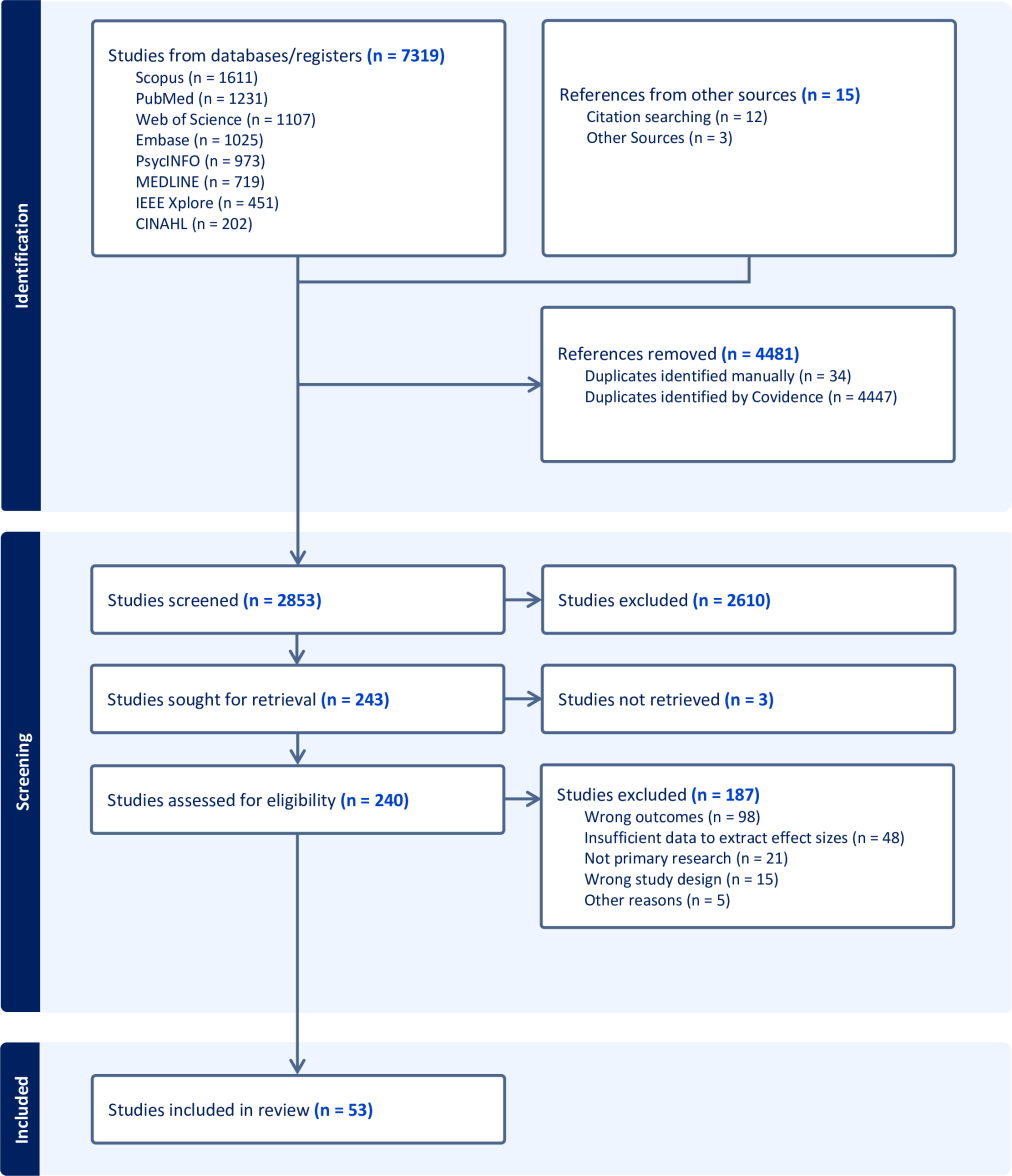
PRISMA flow diagram.

Our search identified 7,319 studies, together with 15 additional studies which were identified from citation searching and other sources (Fig 1). After removing duplicates, we screened the titles and abstracts of 2,853 studies. 2,613 of these were excluded (including three for which we could not obtain a full text version of the article) leaving 240 studies that were assessed for eligibility using full text screening. 187 of these were excluded: 98 because they did not examine suicide or hospital-treated self-harm, 48 because of insufficient data, 21 because they were not primary research, 15 because of the wrong study design, and five for other reasons. This left 53 studies [32–84]. These studies analysed 35 million records and 249,000 occurrences of suicide and self-harm.

The study characteristics are summarised in Table 1. 30 studies were conducted in the United States, five studies in Denmark, five studies in the United Kingdom, three studies in Canada, two studies in South Korea, two studies in Sweden, and one study each in China, France, Iran, the Netherlands, Spain and Turkey. All studies were published between 2015 and 2025 with 44 of these from 2020 onwards.

**Table 1:** Characteristics of included studies.

Source	Country	Population	Data source	Study design	Statistical method	Outcome	Outcome ascertainment	Sample size	Prevalence	Sensitivity	Specificity	LR+	LR-	PPV	NPV
Amini 2016	Iran	People who had attempted suicide.	Survey linked to mortality records	Cohort	Support vector machines	Suicide	>1 year	3,790	8.4%	53.1%	69.9%	1.8	0.7	14.0%	94.2%
Arora 2023	United Kingdom	People aged 18–30 years who were treated as a mental health inpatient.	National register of mental health inpatient records	Cohort	Random forest and ensemble models	Self-harm	>1 year	6,031	4.6%	73.0%	70.3%	2.5	0.4	10.6%	98.2%
Barak-Corren 2017	United States	General hospital patients with three or more inpatient or outpatient visits.	Electronic health records	Cohort	Naive Bayes classifier	Suicide or self-harm	Not reported	864,222	1.2%	33.0%	93.9%	5.4	0.7	6.0%	99.2%
Barak-Corren 2020	United States	General hospital inpatients and outpatients aged 10–90 years with 3 or more visits.	Electronic health records from five independent health systems	Cohort	Naive Bayes classifier	Self-harm	>1 year	1,892,672	1.0%	36.3%	90.0%	3.6	0.7	3.6%	99.3%
Barak-Corren 2023	United States	Patients with multiple sclerosis who are treated in hospital.	Electronic health records	Cohort	Naive Bayes classifier	Suicide or self-harm	>1 year	7,560	0.7%	23.1%	95.0%	4.6	0.8	3.1%	99.4%
Ben-Ari 2015	United States	US veterans who served in the Persian Gulf War.	Veteran Affair’s electronic health record system.	Cohort	Random forest	Self-harm	>1 year	82,500	2.7%	27.4%	99.5%	55.1	0.7	60.4%	98.0%
Bentley 2025	United States	Patients assessed for suicidal risk in inpatient, outpatient and emergency department settings.	Electronic health records	Cohort	Random forest	Self-harm	180 days	812,114	0.4%	69.7%	86.9%	5.3	0.3	2.2%	99.9%
Bittar 2019	United Kingdom	Cases were patients treated for self-harm in acute physical or specialist mental health services. Controls were age-matched patients from the same health service.	Electronic medical records from a single health service	Case-control	Natural language processing	Self-harm	30 days	4,235	20.0%	63.0%	89.9%	6.3	0.4	61.0%	90.7%
Cansel 2023	Turkey	Patients presenting to the emergency department who had attempted suicide and healthy controls.	Patient health records	Case-control	Extreme Gradient Boosting	Self-harm	>1 year	218	45.0%	77.6%	87.5%	6.2	0.3	83.5%	82.7%
Carson 2024	United States	Youth (12–20 years) hospitalised in a psychiatric inpatient unit.	Electronic health record—structured and unstructured data from the adolescent inpatient psychiatry unit of Cambridge Health Alliance in Massachusetts.	Case-cohort	Random forest classifier and a gradient boosting classifier	Self-harm	90 days	558	1.8%	80.0%	76.3%	3.4	0.3	5.8%	99.5%
Chen 2020	Sweden	People aged 18–39 treated as inpatients and outpatients of psychiatric specialty care services.	National registry data	Cohort	Random forest classifier and a gradient boosting classifier	Suicide or self-harm	90 days	108,276	3.7%	47.2%	96.6%	14.0	0.5	34.9%	97.9%
Chen 2024	United States	Cases were patients treated for self-harm in inpatient, outpatient or emergency department settings (aged 18–64 years). Controls were age-sex matched patients from the same settings.	Electronic health records	Case-control	Tree guided feature selection and logic aggregation	Self-harm	>1 year	13,398	9.1%	22.7%	96.8%	7.0	0.8	41.3%	92.6%
Cho 2020	South Korea	General population.	National health insurance data	Cohort	Random forest and bootstrap aggregating	Suicide	1 year	111,843	0.1%	65.7%	78.8%	3.1	0.4	0.3%	100.0%
Cho 2021	South Korea	General population aged 65 and over	Health screening cohort database	Cohort	Random forest	Suicide	Not reported	48,047	0.2%	60.0%	82.2%	3.4	0.5	0.7%	99.9%
Coley 2021	United States	Patients aged 13 years or older who had at least one outpatient mental health specialty visit.	Electronic health records	Cohort	Random forest	Self-harm	90 days	530,639	0.7%	49.1%	92.0%	6.2	0.6	4.0%	99.6%
Coley 2023	United States	Patients aged 13 years or older who had at least one outpatient mental health specialty visit.	Electronic health records and insurance claims data linked with state mortality records	Cohort	Random forest	Suicide	90 days	3,754,137	0.0%	39.2%	100.0%	853.5	0.6	13.9%	100.0%
DelPozo-Banos 2018	United Kingdom	Cases were people who died by suicide. Controls were matched by gender and age from the general population.	Data linkage of mortality, demographic, primary care, hospital and emergency department data.	Case-control	Artificial neural networks	Suicide	>1 year	60,684	4.3%	64.6%	81.9%	3.6	0.4	13.8%	98.1%
Edgcomb 2021a	United States	General hospital patients with serious mental illness (major affective and chronic psychotic disorders).	Electronic health records	Cohort	Classification and regression trees	Self-harm	1 year	780,884	5.2%	73.3%	82.2%	4.1	0.3	18.3%	98.3%
Edgcomb 2021b	United States	Women treated in hospital for mental health.	Electronic health records	Cohort	Classification and regression trees	Self-harm	1 year	15,644	1.4%	83.0%	81.1%	4.4	0.2	5.8%	99.7%
Edgcomb 2023	United States	Children (aged 10–17) treated in emergency departments.	Electronic health records	Case-control	Random forest	Self-harm	Not reported	600	47.3%	85.6%	91.8%	10.4	0.2	90.3%	87.6%
Fernandes 2018	United Kingdom	Psychiatric patients treated in secondary and tertiary care.	Electronic health records	Case-control	Natural language processing	Self-harm	Not reported	500	77.6%	98.2%	29.5%	1.4	0.1	82.8%	82.5%
Gholi Zadeh Kharrat 2024	Canada	Cases were people aged 15 years and older who died by suicide. Controls were a 1% random sample of the Quebec population.	Linked data from five sources: health insurance registry, physician billing database, hospitalisation database, prescription claims database and a vital statistics database.	Case-control	Extreme gradient boosting and multilayer perception	Suicide	>1 year	654,489	1.4%	8.3%	99.9%	99.5	0.9	57.8%	98.8%
Gradus 2020	Denmark	Cases were people who died by suicide. Controls were drawn from the general population.	National registry data	Case-cohort	Classification and regression trees and random forest	Suicide	>1 year	279,286	5.0%	38.0%	96.7%	11.7	0.6	38.3%	96.7%
Gradus 2021	Denmark	Cases were all people who had made suicide attempt. Controls were drawn from the general population.	Nationwide registry data	Case-cohort	Classification and regression trees and random forest	Self-harm	Not reported	288,157	8.0%	43.8%	98.4%	26.7	0.6	69.8%	95.3%
Haroz 2024	United States	Patients treated in an American Indian health service.	Electronic health records from a single Indian Health Service in the Southwestern United States	Cohort	Penalised LASSO	Self-harm	90 days	16,835	1.9%	36.7%	71.9%	1.3	0.9	2.5%	98.3%
Jiang 2021	Denmark	Cases were people discharged from a psychiatric hospital. Controls were drawn from the general population.	National registry data	Case-cohort	Classification and regression trees and random forest	Suicide	30 days	25,764	4.7%	28.5%	96.0%	7.1	0.7	25.9%	96.5%
Jiang 2024	Denmark	People with depression.	National medical and administrative registries in Denmark	Case-cohort	LASSO	Self-harm	>1 year	17,995	33.5%	14.0%	99.6%	38.0	0.9	95.0%	69.7%
Kessler 2020	United States	Veterans who had psychiatric hospital admission	Veteran Health Administration health records linked with mortality data	Case-control	Super learner ensemble machine learning	Suicide	1 year	117,278	0.3%	22.4%	94.9%	4.4	0.8	1.2%	99.8%
Martinez-Romo 2025	Spain	Cases were patients treated for self harm in a psychiatry department of a hospital. Controls were all other psychiatric patients.	Electronic health records including free text notes	Case-control	Guardian-BERT	Self-harm	>1 year	778	18.0%	90.7%	97.0%	30.5	0.1	87.0%	97.9%
Metzger 2017	France	Patients attending the emergency department for any reason.	Electronic health records of structured and unstructured data	Case-control	Naive Bayes	Self-harm	Not reported	390	25.1%	94.9%	99.0%	92.4	0.1	96.9%	98.3%
Nielsen 2023	Denmark	Patients discharged after a psychiatric inpatient stay.	National registry data	Cohort	Gradient boosting and categorical boosting model	Suicide	30 days	180,795	0.1%	52.3%	81.7%	2.9	0.6	0.3%	99.9%
O’Reilly 2024	Sweden	Mental health outpatients aged 9–18 years.	Population-based registries	Cohort	Random forest	Suicide or self-harm	1 year	12,933	0.6%	83.7%	48.0%	1.6	0.3	1.0%	99.8%
Obeid 2020	United States	Cases were patients treated for self-harm in hospital. Controls were patients with no history of self-harm.	Electronic health records	Case-control	Deep neural networks	Self-harm	>1 year	342	50.9%	69.5%	88.7%	6.1	0.3	86.4%	73.8%
Penfold 2021	United States	Patients aged 13 years and <18 years had outpatient visits at mental health clinics.	Electronic health record and administrative claims data	Cohort	LASSO	Suicide or self-harm	90 days	361,176	1.4%	26.5%	95.3%	5.6	0.8	7.2%	98.9%
Sanderson 2020a	Canada	People presenting to the emergency department for self-harm.	Registry data from statewide health system	Case-control	Gradient boosted trees	Suicide	Not reported	39,028	9.1%	69.8%	82.9%	4.1	0.4	29.0%	96.5%
Sanderson 2020b	Canada	Cases were people who died by suicide. Controls were drawn from the general population.	Electronic health records linked with mortality records	Cohort	Gradient boosted trees	Suicide	90 days	33,694	0.8%	68.7%	89.1%	6.3	0.4	4.8%	99.7%
Sheu 2023	United States	Psychiatric emergency department patients	Electronic health records	Cohort	Random survival forests	Self-harm	1 year	13,098	2.3%	29.2%	95.0%	5.9	0.7	12.3%	98.3%
Sheu 2024	United States	All patients treated within a hospital system (consisting of eight hospitals)	Electronic health records	Cohort	Neural ordinary differential equations	Self-harm	>1 year	170,238	0.1%	68.6%	95.0%	13.7	0.3	1.6%	100.0%
Shortreed 2023	United States	Patients with mental health specialty visits.	Electronic health records linked to state mortality data	Cohort	Ensemble models	Suicide	90 days	4,574,921	0.0%	13.6%	99.0%	13.1	0.9	0.3%	100.0%
Simon 2018	United States	Patients aged 13 or older with mental health specialty visits.	Electronic health records linked to state mortality data	Cohort	LASSO	Suicide	90 days	3,596,725	0.0%	48.1%	95.6%	11.0	0.5	0.3%	100.0%
Simon 2024a	United States	Patients aged 11 or older who visited specialty mental health clinicians.	Insurance claim data and electronic health records	Cohort	LASSO and random forest models	Suicide or self-harm	90 days	4,753,514	0.6%	42.2%	95.3%	8.9	0.6	5.3%	99.6%
Simon 2024b	United States	Patients attended emergency department for mental healthcare.	Electronic health records with suicide death recorded and insurance claims	Cohort	Random forest	Suicide	90 days	2,069,170	0.0%	34.8%	95.0%	7.0	0.7	0.3%	100.0%
Su 2020	United States	Patients aged 10–18 attending emergency department for any reason.	Electronic health records	Cohort	Logistic regression classifier with a sequential forward selection procedure	Self-harm	1 year	8,366	0.7%	38.3%	94.7%	7.3	0.7	5.0%	99.5%
Tsui 2021	United States	Cases were patients attending emergency departments or inpatient units for suicide attempts for the first time. Controls were patients without a history of suicide attempts.	Electronic health records	Case-control	Natural language processing and ensemble of extreme gradient boosting	Self-harm	90 days	11,079	8.8%	95.0%	69.9%	3.2	0.1	23.2%	99.3%
van Mens 2020	Netherlands	Cases were patients attending general practice appointments for attempted suicide. Controls were patients attending general practice appointments for psychological treatment.	Electronic health records	Case-control	Random forest	Self-harm	Not reported	53,822	0.3%	39.1%	97.6%	16.2	0.6	4.6%	99.8%
Walsh 2017	United States	Cases were patients who had made a suicide attempt. Controls were patients with self-injury ICD codes but who had not made an attempt (e.g., unintentional drug overdose, accidental injury, non-suicidal self-injury, injury with unclear intent).	Electronic health records	Case-control	Random forest	Self-harm	1 year	5,167	62.9%	98.3%	58.2%	2.3	0.0	79.9%	95.2%
Wang 2023	United Kingdom	Cases were UK Biobank participants who had attempted or died by suicide. Controls were randomly selected living participants from the biobank with no recent suicidal behaviour.	UK Biobank data	Case-cohort	Light gradient-boosting machine	Suicide or self-harm	1 year	4,683	4.8%	57.8%	95.1%	11.8	0.4	37.2%	97.8%
Wilimitis 2022	United States	General hospital patients aged ≥18 years.	Electronic health records	Cohort	Ensemble models	Self-harm	180 days	120,398	0.4%	58.6%	95.0%	11.8	0.4	4.8%	99.8%
Xu 2020	China	Cases were patients aged > 10 years with at least one self-harm diagnosis. Controls were patients aged > 10 years without a self-harm diagnosis.	Electronic health records	Case-control	Diagnosis to vector (Dx2vec) and deep neural network	Self-harm	1 year	8,149	5.7%	72.0%	96.3%	19.4	0.3	54.0%	98.3%
Xu 2022	United States	Children, adolescents and young adults (10–24 years) with at least one non-suicide related hospitalisation.	Insurance claims database linked with inpatient data	Case-control	Targeted fusion learning framework	Self-harm	Not reported	3,930	1.2%	65.3%	94.7%	12.2	0.4	13.4%	99.5%
Yang 2025	United States	Veterans treated as psychiatric inpatients.	Electronic health records linked with the National Death Index	Cohort	TransformEHR	Suicide	180 days	126,800	0.1%	45.2%	96.3%	12.3	0.6	1.6%	99.9%
Zang 2024	United States	Patients aged 10–24 years in three hospitals systems.	Electronic health records	Cohort	Regularised logistic regression	Self-harm	>1 year	57,055	1.2%	59.1%	90.0%	5.9	0.5	6.8%	99.4%
Zheng 2020	United States	General hospital patients.	Electronic health records	Cohort	Deep neural networks	Self-harm	1 year	112,095	0.2%	6.9%	99.9%	62.2	0.9	10.1%	99.8%

Note: Sample sizes represent the sample in which diagnostic accuracy statistics were calculated. If a study separated the data into training and validation samples, the sample size will be from the validation sample.

Thirty-six studies used a retrospective cohort design and 17 used a case-control design. Thirteen studies predicted suicide, 30 predicted hospital-treated self-harm, 7 predicted suicide/self-harm and three studies developed algorithms separately for suicide and self-harm. The time frame over which these predictions were made were 30 days (3 studies), 90 days (12 studies), 180 days (3 studies), 1 year (11 studies) and >1 year (15 studies). In nine studies, the prediction window was not reported. There was considerable variation in the study populations. Twenty-four studies developed their algorithms in general population or general patient populations. Twenty-two studies developed algorithms in patients treated for psychiatric problems. Six studies developed algorithms in patients presenting to hospital for self-harm or with a history of self-harm. One study used data from another population (patients with multiple sclerosis). The data were predominately drawn from electronic health records, insurer claims data and registry data. The studies used a variety of machine learning methods, including random forests (10 studies), gradient boosted trees (8 studies), classification and regression trees (5 studies), LASSO models (5 studies) naive Bayes classifiers (3 studies) and ensemble learning (3 studies).

The findings of nine studies were reported using TRIPOD or STARD guidelines. Of the 31 items in the TRIPOD checklist, three items were judged to be not relevant for most studies and were removed from the quality assessment. Of the remaining 28 items, the mean number of checklist items that were adhered to across the studies was 20. Checklist items with low adherence were explaining how the sample size had been arrived at (11 studies), describing how missing data was handled (19 studies), reporting unadjusted associations between candidate predictors and the outcome (10 studies) and providing details about how the risk groups were created (11 studies) (S1 Table).

**Fig 2:**
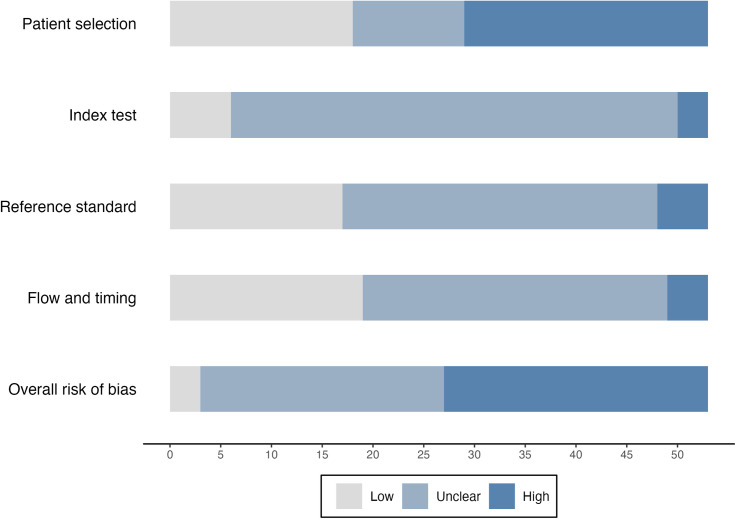
Risk of bias assessments.

For patient selection, 18 studies were judged as having low risk of bias, 24 were at high risk of bias with the remaining 11 studies at unclear risk of bias (Fig 2 and S2 Table). For choice of index test, 6 studies were at low risk of bias, three were at high risk of bias and 44 studies were at unclear risk of bias. For the reference standard, 17 studies were at low risk of bias, 5 studies were at high risk of bias and 31 studies were at unclear risk of bias. For flow and timing of patients, 19 studies were at low risk of bias, 4 were at high risk of bias and 30 studies were at unclear risk of bias. Overall, three studies were judged to be at low risk of bias, 26 studies at high risk of bias, and 24 studies at unclear risk of bias.

The pooled AUROCs ranged from 0.69 to 0.93 with the lowest value for the prediction of suicide from cohort studies and the highest for predicting self-harm from case-control studies (Table 2). The pooled sensitivities ranged from 45% to 82% and the specificities from 91% to 95%. The LR+ values ranged from 6.5 to 9.9 and the LR− from 0.2 to 0.6. Using baseline 1-year prevalence values of 0.7% for suicide, 2.1% for self-harm and 1.6% for suicide/self-harm, the positive predictive values were 6% for suicide (in cohort studies), 16% and 17% for self-harm (in case-control and cohort studies, respectively) and 9% for suicide/self-harm (in cohort studies). The corresponding negative predictive values were 99% for suicide, 90% and 96% for self-harm and 97% for suicide/self-harm.

**Table 2:** Pooled diagnostic accuracy statistics of machine learning instruments to predict suicide, self-harm and suicide/self-harm.

Outcome	Study design	Number of estimates	AUROC	Sensitivity, % (95% CI)	Specificity, % (95% CI)	LR+, (95% CrI)	LR−, (95% CrI)	Positive predictive value, % (95% CrI)	Negative predictive value, % (95% CrI)
Suicide									
	Case-control	4	--	--	--	--	--	--	--
	Cohort	12	0.69	45 (35, 55)	95 (87, 98)	9.9 (3.8, 21.9)	0.6 (0.5, 0.7)	6 (3, 14)	99 (99, 99)
Self-harm									
	Case-control	13	0.93	82 (66, 92)	91 (82, 96)	9.2 (4.8, 16.2)	0.2 (0.1, 0.4)	16 (9, 26)	90 (81, 94)
	Cohort	20	0.80	46 (35, 58)	95 (90, 97)	9.4 (5.6, 14.8)	0.6 (0.5, 0.7)	17 (11, 24)	96 (95, 97)
Suicide/self-harm									
	Case-control	0	--	--	--	--	--	--	--
	Cohort	7	0.80	46 (29, 63)	93 (84, 97)	6.5 (3.6, 11.0)	0.6 (0.4, 0.7)	9 (5, 15)	97 (96, 98)

Notes: Analyses only undertaken when there are at least five studies available for meta-analysis. All pooled sensitivity and specificity estimates calculated using the binomial-normal model that jointly estimates both (logit transformed) pooled values and adjusts for the negative correlation between them. Pooled LR values calculated by sampling sensitivity and specificity values from the bivariate normal distribution using a Monte Carlo Markov chain with 100,000 samples. Positive and negative predictive values calculated using Bayes rule with the following baseline prevalence estimates (suicide 0.7%, self-harm 2.1%, suicide/self-harm 1.6%). CI, confidence intervals. CrI, credible intervals.

Table 3 shows the positive predictive values for a hypothetical algorithm with LR+ values ranging from 1 to 50 alongside indicative 1-year probabilities of suicidal behaviours in different populations. In a low prevalence population, for example, predicting suicide in the general population (baseline prevalence 0.01% per year [30]), in the LR+ values we observed (LR+ = 6–10), the positive predictive values ranged from 0.06% to 0.10%. In a medium prevalence population, for example, predicting suicide after discharge from hospital for the treatment of self-harm (baseline prevalence 2% per year [29]), positive predictive values ranged from 11% to 17%. In a high prevalence population, for example, predicting self-harm after discharge from hospital for self-harm (baseline prevalence 16% per year [29]), the positive predictive values ranged from 53% to 66%. Positive predictive values improved at higher LR+ values than we observed in our pooled analysis. At LR+ = 20, the positive predictive value was 29% in a medium prevalence population (79% in a high prevalence population), and at LR+ = 50, it was 51% (91% in a high prevalence population).

**Table 3:** Positive predictive values by 1-year base prevalences and positive likelihood ratios.

Base prevalence	0.01%	0.5%	1.5%	2.0%	6.5%	16%
**Example outcome and population**	Suicide in general population	Suicide after discharge from inpatient psychiatric facility	Self-harm in general population	Suicide after discharge for self-harm	Self-harm after discharge from psychiatric facility	Self-harm after discharge for self-harm
**LR+**	**Positive predictive values**					
1	0.01%	0.5%	1.5%	2.0%	6.5%	16.0%
2	0.02%	1.0%	3.0%	3.9%	12.2%	27.6%
3	0.03%	1.5%	4.4%	5.8%	17.3%	36.4%
4	0.04%	2.0%	5.7%	7.5%	21.8%	43.2%
5	0.05%	2.5%	7.1%	9.3%	25.8%	48.8%
6	0.06%	2.9%	8.4%	10.9%	29.4%	53.3%
7	0.07%	3.4%	9.6%	12.5%	32.7%	57.1%
8	0.08%	3.9%	10.9%	14.0%	35.7%	60.4%
9	0.09%	4.3%	12.1%	15.5%	38.5%	63.2%
10	0.10%	4.8%	13.2%	16.9%	41.0%	65.6%
15	0.15%	7.0%	18.6%	23.4%	51.0%	74.1%
20	0.20%	9.1%	23.3%	29.0%	58.2%	79.2%
50	0.50%	20.1%	43.2%	50.5%	77.7%	90.5%

Note: LR+ is defined as sensitivity/(1 − specificity). Shaded region refers to the range of pooled LR+ values observed in the meta-analysis.

The sROCs are contained in S1 Fig. The figure shows that the estimates are generally clustered close together on the two dimensions (sensitivity and the false positive rate). *I*^2^ estimates ranged from 1.6% to 11.4%.

## Discussion

In this systematic review and meta-analysis of algorithms developed using machine learning tools to predict suicidal behaviour, we found these algorithms had good accuracy when assessed using a global measure, the area under the curve, but poor accuracy when assessed against more clinically relevant individual measures. We found that the algorithms had modest sensitivity and high specificity. This combination of sensitivity and specificity meant that while the algorithms are good at identifying people who will *not* re-present for self-harm or die by suicide, they are generally poor at identifying those who will. The modest sensitivity observed in the cohort studies indicates that more than half of those who repeat self-harm or die by suicide are misclassified as low risk.

The sensitivity and specificity values we observed translate into LR+ values that are just under the clinically meaningful minimal threshold of LR+ ≥ 10 [85]. However, the low baseline prevalence of suicidal behaviour, taken either from the cohort studies included in our review or from externally derived prevalence estimates [26–30], meant that the positive predict values of these algorithms were also very low. To illustrate, the in-sample positive predictive values were 6% for suicide, 16%—17% for self-harm and 9% for suicide/self-harm. When an LR+ value of 10 was applied to low, medium and high prevalence populations, the positive predictive values were 0.10%, 17% and 66%. The only theoretical scenarios where the positive predictive values were high enough to be clinically useful would be when the LR+ was ≥50 and the base prevalence was ≥6.5% per year (equivalent to an event rate of 6,500 per 100,000 person years) or when LR+ was ≥20 and the base prevalence was ≥16% per year (16,000 per 100,000 person years). These high positive predictive values are unlikely to be realised in real-world clinical settings for two reasons. First, predictions in high prevalence populations, such as in patients who have been discharged from a psychiatric facility or received treatment in hospital for self-harm, will be most clinically useful over a shorter window than the 1-year prevalence estimates used here (for example, 24 or 48 h after discharge through to 30 days). Prevalence will therefore be much lower than the values we used, and consequently, the positive predictive values will also be lower. To illustrate, while the 1-year baseline probability of self-harm after discharge from a psychiatric facility is 6.5%, the 4-week estimate is only 2.1% [28]. At this value, the positive predictive value is only 30% for an LR+ value of 20. Second, it is difficult to develop an algorithm with high LR+ values as it requires identifying a threshold with both high sensitivity and high specificity. In practice, there is a trade-off between sensitivity and specificity. Finding a threshold that increases the value of one of these measures will result in decreasing the value of the other. Most ways of getting a high LR+ value require very high specificity values (≥97%) which means sensitivity is likely to be corresponding low, leading to most cases being misclassified as low risk.

There appear to be two reasons for the recent focus on the development of new algorithms to predict suicide and self-harm. One reason is to screen for risk of suicide and self-harm [34,36,41,44,45,48,50,56,71,72,76,79,81,82,84]. In this model, the algorithm flags high-risk patients in the electronic medical record and these patients then undergo a further risk assessment. Most studies have focussed on this two-stage process being applied to psychiatric inpatients and outpatients, but it has also been suggested that this be applied to general practice patients [76]. If these algorithms are to be used for automatically screening medical records then they should meet the criteria set down for a viable, effective and appropriate screening programme [86]. Yet against the 12 consolidated screening principles [87] the algorithms for suicide and self-harm appear to meet one criterion: the epidemiology of the disease or condition is adequately understood; and another partially: that there is an agreed-upon course of action for screening participants with a positive test result. The algorithms do not appear to meet the other criteria, namely: the natural history of the disease or condition is clearly understood; the target population for screening is clearly defined; the screening test has sufficient performance characteristics; the screening test results are clearly interpretable; there is adequate infrastructure to allow for timely access to all components of the screening programme; the screening programme is coordinated with the broader healthcare system; the screening programme is acceptable and ethical; the overall benefits of the screening programme outweigh the harms; the full costs of the screening programme have been assessed in an economic evaluation; and the screening programme has clear goals and it is evaluated against these goals. On this basis, none of the algorithms we studied appear to be suitable as a screening tool for suicide or self-harm in unselected clinical populations.

The second reason machine learning algorithms have been developed is to prioritise the highest risk individuals for expensive or high-intensity interventions (for example, psychiatric hospitalisation or intensive case management by psychiatric services after discharge) [59,64,66,78,88]. One illustrative study found that among those with mental health speciality visits, those in the top 5% of risk accounted for 43% of suicide attempts and 48% of suicides over a 90-day prediction window [71]. The problem with this approach is that it results in algorithms with modest sensitivity and poor positive predictive values [3,89]. As the threshold that defines a positive test result is raised, the number of cases of suicide or self-harm detected by the algorithm (the true positives) decreases and the number of undetected cases increases (the false negatives). At a very high threshold (for example, the top 5% of risk continuum), it is likely that the number of undetected cases outnumber the detected cases (i.e., sensitivity will be <50%). Regarding the specificity, increasing the threshold will benefit the specificity of the algorithms because the number of non-cases that fall below the threshold (the true negatives) will increase. This is the pattern of results we see in our meta-analysis. The pooled sensitivities were generally below 50% and the specificities above 90%, and when combined with the low case prevalence, meant the positive predictive values were very low (because of the large proportion of false positives). The implication of using a high threshold to allocate treatment is that most cases of suicide and self-harm will be misclassified as low risk, and most people who test positive will receive an intervention they may not need. In other words, these intensive and expensive services will largely be delivered to the wrong people.

One argument in favour of using risk prediction algorithms is that it may be a cost-effective way of allocating expensive interventions. This strategy could be appealing to third-party payers and public health providers in an environment where healthcare resources are scarce. Some research has examined the circumstances under which a suicide risk prediction test might be cost-effective [88]. In a simulation study, the authors found an active contact and follow-up intervention could be cost-effective when people were allocated to this intervention using a test with sensitivity of 17% or greater when specificity was 95%. Similarly, the same study showed cognitive behavioural therapy could be cost-effective when the test used for allocation had sensitivity of 36% or greater when specificity was 95%. However, there are important caveats to these findings. First, the low sensitivity implies that a high threshold was being used to allocate treatment in these simulations, but a high threshold means that only a small number of people are allocated to these interventions. Second, and as discussed above, when sensitivity is less than 50%, there are more undetected cases at that threshold than detected cases. Many of these people could have benefited from the intervention, but would not have received it as they scored below the threshold on the risk assessment.

The diagnostic accuracy of machine learning algorithms for suicide and self-harm is similar to that of traditional risk assessment instruments [7,9]. The poor accuracy of these traditional instruments was one of the factors that led to clinical guidelines in several countries recommending that risk stratification not be undertaken in order to allocate aftercare services and that alternatives, such a needs-based psychosocial assessment be offered to patients instead in order to foster and focus aftercare interventions [10,12]. The National Institute of Clinical and Health Care Excellence guidelines [12] recommend that after an episode of self-harm, a mental health professional should carry out a psychosocial assessment to develop a therapeutic relationship with the patient and a shared understanding of why they have self-harmed, undertake needs assessment, ensure the patient is offered the care they need, and give family and carers information about the patient’s condition and diagnosis. Ideally, mental health clinicians should develop a therapeutic alliance with the patient that is organised around four components: predisposing factors (their history of self-harm, mental health and other relevant events), modifiable factors (things that are changeable, such as relationship issues, substance use, mood and mental health and access to means), future factors (anticipated events such as anniversaries, discharge from hospital or criminal proceedings) and protective factors (problem-solving skills, social and family support, engagement with services, insight and hope) [90]. Given that machine learning algorithms, including those that use dynamic risk formulation [68,70,77], appear to be no better at predicting suicide or self-harm than traditional risk assessment instruments, we see no compelling new evidence to warrant a change to these guidelines.

More generally, there are a number of effective aftercare interventions suitable for people presenting to hospital for self-harm that can be applied without first undertaking risk stratification to determine the allocation of treatment. Examples of interventions that have been shown to be effective for reducing the repetition of self-harm include psychological and psychosocial interventions (e.g., cognitive behavioural therapy or interventions with an interpersonal focus [4,91], brief contact interventions [92], multilevel interventions for the reduction of suicide and suicide attempts in clinical populations [6], and safety planning interventions [93]. All these interventions have financial and non-financial costs associated with them, and decisions about whether to deploy them in a hospital setting should be made with due consideration of whether the intrusiveness, burdensomeness and ethicality are proportionate to the benefits. Finally, we are concerned that the focus on risk assessment can be falsely reassuring and a distraction from the delivery of basic clinical services like ensuring all patients who present to the emergency department for self-harm are seen in a timely manner, are properly assessed and receive appropriate follow-up care [94]. In the UK and Australian context, this is apparent in the concerns of patients and service users about impersonal tick boxes rather than holistic assessments [95] and clinicians or health services being preoccupied with potential blame rather than delivering high-quality care [96,97].

Instead of predicting suicide and self-harm, there may be other ways in which artificial intelligence could be used to contribute to better outcomes for suicidal patients. Future research could consider how machine learning methods could be used to augment existing collaborative psychosocial assessments. Specifically, can machine learning methods be used to identify modifiable risk factors for suicide and self-harm for individual patients? This may be a more tractable problem as the prevalence of many risk factors is likely to be higher than the prevalence of suicide or self-harm. If such modelling can be done, then there are interesting follow-on questions about the acceptability of this approach for patients and clinical staff, and whether such an approach is superior to gathering information directly from patients and caregivers. Another interesting question for future research is to consider how artificial intelligence could be used to inform clinical decision support tools. This is distinct from using a risk classification to allocate treatment; rather, it is a question about whether artificial intelligence, when combined with information about an individual patient, can make suggestions for treatment pathways. Some work on this has been undertaken in other areas of medicine (e.g., in oncology to optimise drug dosage for individual patients [98]), and it is an open question as to whether this approach can be applied to the treatment of individuals with psychiatric symptoms and disorders.

Our study has a number of strengths. We used a broad set of search terms to capture studies that have used machine learning to predict suicidal behaviour. We searched eight databases that comprised a wide range of disciplines (e.g., medicine, psychology, health sciences, engineering and computer sciences). We focussed only on suicide and hospital-treated self-harm as the outcomes, not self-reported behaviour or scores on an instrument. The included studies use a variety of different machine learning methods. We were able to assess the quality of the literature, and we showed that at least half the studies on this topic are at high risk of bias and a substantial number are at unclear risk of bias. We were able to examine a range of diagnostic accuracy statistics, and we were able to recalibrate case-control studies to estimate positive and negative predictive values using the prevalence from cohort studies. Finally, we were able to estimate the positive predictive values for different outcomes (suicide or self-harm) in different populations (the general population, psychiatric patients, patients treated for self-harm).

Against this, our study had limitations. We had to exclude 48 studies because they did not present sufficient information for data extraction. The period over which follow-up outcome data were gathered varied between studies, from 30 days to 2 years for the majority of studies. Most of the included studies were judged to be at high or unclear risk of bias. We were unable to estimate pooled values for two groups of studies: case-control studies of suicide and case-control studies of suicide/self-harm. We were unable to assess publication bias as tools have not been developed to assess publication bias of diagnostic and accuracy studies. We were unable to assess the potential biases in individual algorithms. Finally, a number of studies used data collected from the same health system or data-linkage system. We were unable to adjust for this in our analyses.

In conclusion, our systematic review and meta-analysis has shown that algorithms developed using machine learning tools to predict suicide and self-harm suffer from the same problems as the traditional risk scales used to predict suicidal behaviour. The algorithms have modest sensitivity and low positive predictive values, resulting in most cases of suicide or self-harm occurring amongst those classified as low risk, and a large proportion of false positives in those classified as high risk.

## Supporting information

S1 PRISMA Checklist PRISMA 2020 Checklist. This checklist is licensed under the Creative Commons Attribution 4.0 International License (CC BY 4.0; https://creativecommons.org/licenses/by/4.0/). (S1_PRISMA_Checklist.DOCX)

S1 Table TRIPOD checklist adherence for 53 included studies. (S1_Table.DOCX)

S2 Table Risk of bias ratings for each study. (S2_Table.DOCX)

S1 Fig sROC curves of machine learning instruments to predict suicide, self-harm, suicide/self-harm. (DOCX)

## Abbreviations

AUROC: area under the receiver operating characteristic curve
QUADAS 2: Quality Assessment of Diagnostic Accuracy Studies instrument
sROC: summary receiver operating characteristic curves
STARD: Standards for Reporting of Diagnostic Accuracy Studies
TRIPOD: Transparent Reporting of a Multivariable Prediction Model for Individual Prognosis or Diagnosis

## References

[1] Franklin JC, Ribeiro JD, Fox KR, Bentley KH, Kleiman EM, Huang X, et al. Risk factors for suicidal thoughts and behaviors: a meta-analysis of 50 years of research. Psychol Bull. 2017;143(2):187–232. doi: 10.1037/bul0000084 27841450

[2] Ribeiro JD, Franklin JC, Fox KR, Bentley KH, Kleiman EM, Chang BP, et al. Self-injurious thoughts and behaviors as risk factors for future suicide ideation, attempts, and death: a meta-analysis of longitudinal studies. Psychol Med. 2016;46(2):225–36. doi: 10.1017/S0033291715001804 26370729 PMC4774896

[3] Carter G, Spittal MJ. Suicide risk assessment. Crisis. 2018;39(4):229–34. doi: 10.1027/0227-5910/a000558 29972324

[4] Hetrick SE, Robinson J, Spittal MJ, Carter G. Effective psychological and psychosocial approaches to reduce repetition of self-harm: a systematic review, meta-analysis and meta-regression. BMJ Open. 2016;6(9):e011024. doi: 10.1136/bmjopen-2016-011024 27660314 PMC5051331

[5] DeCou CR, Comtois KA, Landes SJ. Dialectical behavior therapy is effective for the treatment of suicidal behavior: a meta-analysis. Behav Ther. 2019;50(1):60–72. doi: 10.1016/j.beth.2018.03.009 30661567

[6] Hofstra E, van Nieuwenhuizen C, Bakker M, Özgül D, Elfeddali I, de Jong SJ, et al. Effectiveness of suicide prevention interventions: a systematic review and meta-analysis. Gen Hosp Psychiatry. 2020;63:127–40. doi: 10.1016/j.genhosppsych.2019.04.011 31078311

[7] Carter G, Milner A, McGill K, Pirkis J, Kapur N, Spittal MJ. Predicting suicidal behaviours using clinical instruments: Systematic review and meta-analysis of positive predictive values for risk scales. Br J Psychiatry. 2017;210(6):387–95. doi: 10.1192/bjp.bp.116.182717 28302700

[8] Large M, Kaneson M, Myles N, Myles H, Gunaratne P, Ryan C. Meta-analysis of longitudinal cohort studies of suicide risk assessment among psychiatric patients: heterogeneity in results and lack of improvement over time. PLoS One. 2016;11(6):e0156322. doi: 10.1371/journal.pone.0156322 27285387 PMC4902221

[9] Quinlivan L, Cooper J, Meehan D, Longson D, Potokar J, Hulme T, et al. Predictive accuracy of risk scales following self-harm: multicentre, prospective cohort study. Br J Psychiatry. 2017;210(6):429–36. doi: 10.1192/bjp.bp.116.189993 28302702 PMC5451643

[10] Carter G, Page A, Large M, Hetrick S, Milner AJ, Bendit N, et al. Royal Australian and New Zealand College of Psychiatrists clinical practice guideline for the management of deliberate self-harm. Aust N Z J Psychiatry. 2016;50(10):939–1000. doi: 10.1177/0004867416661039 27650687

[11] LeFevre ML, U.S. Preventive Services Task Force. Screening for suicide risk in adolescents, adults, and older adults in primary care: U.S. Preventive Services Task Force recommendation statement. Ann Intern Med. 2014;160(10):719–26. doi: 10.7326/M14-0589 24842417

[12] National Institute for Health and Care Excellence. Self-harm: assessment, management and preventing recurrence. National Institute for Health and Care Excellence, 2022. 36595613

[13] The Joint Commission. R3 Report National patient safety goal for suicide prevention; 2019. [cited 4 Jan 2025]. Available from: https://www.jointcommission.org/

[14] Kirtley OJ, van Mens K, Hoogendoorn M, Kapur N, de Beurs D. Translating promise into practice: a review of machine learning in suicide research and prevention. Lancet Psychiatry. 2022;9(3):243–52. doi: 10.1016/S2215-0366(21)00254-6 35183281

[15] Lijmer JG, Mol BW, Heisterkamp S, Bonsel GJ, Prins MH, van der Meulen JH, et al. Empirical evidence of design-related bias in studies of diagnostic tests. JAMA. 1999;282(11):1061–6. doi: 10.1001/jama.282.11.1061 10493205

[16] Akobeng AK. Understanding diagnostic tests 1: sensitivity, specificity and predictive values. Acta Paediatr. 2007;96(3):338–41. doi: 10.1111/j.1651-2227.2006.00180.x 17407452

[17] Page MJ, Moher D, Bossuyt PM, Boutron I, Hoffmann TC, Mulrow CD, et al. PRISMA 2020 explanation and elaboration: updated guidance and exemplars for reporting systematic reviews. BMJ. 2021;372:n160. doi: 10.1136/bmj.n160 33781993 PMC8005925

[18] Collins GS, Reitsma JB, Altman DG, Moons KGM. Transparent reporting of a multivariable prediction model for individual prognosis or diagnosis (TRIPOD): the TRIPOD statement. BMJ. 2015;350:g7594. doi: 10.1136/bmj.g7594 25569120

[19] Cohen JF, Korevaar DA, Altman DG, Bruns DE, Gatsonis CA, Hooft L, et al. STARD 2015 guidelines for reporting diagnostic accuracy studies: explanation and elaboration. BMJ Open. 2016;6(11):e012799. doi: 10.1136/bmjopen-2016-012799 28137831 PMC5128957

[20] Whiting PF, Rutjes AWS, Westwood ME, Mallett S, Deeks JJ, Reitsma JB, et al. QUADAS-2: a revised tool for the quality assessment of diagnostic accuracy studies. Ann Intern Med. 2011;155(8):529–36. doi: 10.7326/0003-4819-155-8-201110180-00009 22007046

[21] Doebler P. Mada: meta-analysis of diagnostic accuracy. 2022.

[22] Reitsma JB, Glas AS, Rutjes AWS, Scholten RJPM, Bossuyt PM, Zwinderman AH. Bivariate analysis of sensitivity and specificity produces informative summary measures in diagnostic reviews. J Clin Epidemiol. 2005;58(10):982–90. doi: 10.1016/j.jclinepi.2005.02.022 16168343

[23] Holling H, Böhning W, Masoudi E, Böhning D, Sangnawakij P. Evaluation of a new version of I2 with emphasis on diagnostic problems. Commun Stat. 2019;49(4):942–72. doi: 10.1080/03610918.2018.1489553

[24] Zwinderman AH, Bossuyt PM. We should not pool diagnostic likelihood ratios in systematic reviews. Stat Med. 2008;27(5):687–97. doi: 10.1002/sim.2992 17611957

[25] Zhou XH, Obuchowski NA, McClish DK. Statistical methods in diagnostic medicine. John Wiley & Sons. 2014.

[26] Carr MJ, Ashcroft DM, Kontopantelis E, Awenat Y, Cooper J, Chew-Graham C, et al. The epidemiology of self-harm in a UK-wide primary care patient cohort, 2001-2013. BMC Psychiatry. 2016;16:53. doi: 10.1186/s12888-016-0753-5 26923884 PMC4770684

[27] Chung DT, Ryan CJ, Hadzi-Pavlovic D, Singh SP, Stanton C, Large MM. Suicide rates after discharge from psychiatric facilities: a systematic review and meta-analysis. JAMA Psychiatry. 2017;74(7):694–702. doi: 10.1001/jamapsychiatry.2017.1044 28564699 PMC5710249

[28] Gunnell D, Hawton K, Ho D, Evans J, O’Connor S, Potokar J, et al. Hospital admissions for self harm after discharge from psychiatric inpatient care: cohort study. BMJ. 2008;337:a2278. doi: 10.1136/bmj.a2278 19018041 PMC2590882

[29] Owens D, Horrocks J, House A. Fatal and non-fatal repetition of self-harm. Systematic review. Br J Psychiatry. 2002;181:193–9. doi: 10.1192/bjp.181.3.193 12204922

[30] World Health Organization. Suicide worldwide in 2019: global health estimates. Geneva: World Health Organization; 2021. [cited 11 Oct 2024]. Available from: https://iris.who.int/bitstream/handle/10665/341728/9789240026643-eng.pdf?sequence=1

[31] Viechtbauer V. Meta-analysis package for R. 2024.

[32] Amini P, Ahmadinia H, Poorolajal J, Moqaddasi Amiri M. Evaluating the high risk groups for suicide: a comparison of logistic regression, support vector machine, decision tree and artificial neural network. Iran J Public Health. 2016;45(9):1179–87. 27957463 PMC5149472

[33] Arora A, Bojko L, Kumar S, Lillington J, Panesar S, Petrungaro B. Assessment of machine learning algorithms in national data to classify the risk of self-harm among young adults in hospital: a retrospective study. Int J Med Inform. 2023;177:105164. doi: 10.1016/j.ijmedinf.2023.105164 37516036

[34] Barak-Corren Y, Castro VM, Javitt S, Hoffnagle AG, Dai Y, Perlis RH, et al. Predicting suicidal behavior from longitudinal electronic health records. Am J Psychiatry. 2017;174(2):154–62. doi: 10.1176/appi.ajp.2016.16010077 27609239

[35] Barak-Corren Y, Castro VM, Javitt S, Nock MK, Smoller JW, Reis BY. Improving risk prediction for target subpopulations: predicting suicidal behaviors among multiple sclerosis patients. PLoS One. 2023;18(2):e0277483. doi: 10.1371/journal.pone.0277483 36795700 PMC9934377

[36] Barak-Corren Y, Castro VM, Nock MK, Mandl KD, Madsen EM, Seiger A, et al. Validation of an electronic health record-based suicide risk prediction modeling approach across multiple health care systems. JAMA Netw Open. 2020;3(3):e201262. doi: 10.1001/jamanetworkopen.2020.1262 32211868 PMC11136522

[37] Ben-Ari A, Hammond K, editors. Text mining the EMR for modeling and predicting suicidal behavior among US Veterans of the 1991 Persian Gulf War. 48th Hawaii International Conference on System Sciences, Kauai, HI, USA; 2015.

[38] Bentley KH, Kennedy CJ, Khadse PN, Brooks Stephens JR, Madsen EM, Flics MJ, et al. Clinician suicide risk assessment for prediction of suicide attempt in a large health care system. JAMA Psychiatry. 2025;82(6):599–608. doi: 10.1001/jamapsychiatry.2025.0325 40202745 PMC11983293

[39] Bittar A, Velupillai S, Roberts A, Dutta R. Text classification to inform suicide risk assessment in electronic health records. MEDINFO 2019. The 17th World Congress on Medical and Health Informatics. Lyon, France: IOS Press; 2019. p. 40–4. 10.3233/SHTI190179 31437881

[40] Cansel N, Yagin FH, Akan M, Ilkay Aygul B. Interpretable estimation of suicide risk and severity from complete blood count parameters with explainable artificial intelligence methods. Psychiatr Danub. 2023;35(1):62–72. doi: 10.24869/psyd.2023.62 37060594

[41] Carson NJ, Yang X, Mullin B, Stettenbauer E, Waddington M, Zhang A, et al. Predicting adolescent suicidal behavior following inpatient discharge using structured and unstructured data. J Affect Disord. 2024;350:382–7. doi: 10.1016/j.jad.2023.12.059 38158050 PMC10923087

[42] Chen J, Aseltine RH, Wang F, Chen K. Tree-guided rare feature selection and logic aggregation with electronic health records data. J Am Stat Assoc. 2024;119(547):1765–77. doi: 10.1080/01621459.2024.2326621

[43] Chen Q, Zhang-James Y, Barnett EJ, Lichtenstein P, Jokinen J, D’Onofrio BM, et al. Predicting suicide attempt or suicide death following a visit to psychiatric specialty care: a machine learning study using Swedish national registry data. PLoS Med. 2020;17(11):e1003416. doi: 10.1371/journal.pmed.1003416 33156863 PMC7647056

[44] Cho S-E, Geem ZW, Na K-S. Prediction of suicide among 372,813 individuals under medical check-up. J Psychiatr Res. 2020;131:9–14. doi: 10.1016/j.jpsychires.2020.08.035 32906052

[45] Cho S-E, Geem ZW, Na K-S. Development of a suicide prediction model for the elderly using health screening data. Int J Environ Res Public Health. 2021;18(19):10150. doi: 10.3390/ijerph181910150 34639457 PMC8507921

[46] Coley RY, Liao Q, Simon N, Shortreed SM. Empirical evaluation of internal validation methods for prediction in large-scale clinical data with rare-event outcomes: a case study in suicide risk prediction. BMC Med Res Methodol. 2023;23(1):33. doi: 10.1186/s12874-023-01844-5 36721082 PMC9890785

[47] Coley RY, Walker RL, Cruz M, Simon GE, Shortreed SM. Clinical risk prediction models and informative cluster size: assessing the performance of a suicide risk prediction algorithm. Biom J. 2021;63(7):1375–88. doi: 10.1002/bimj.202000199 34031916 PMC9134927

[48] DelPozo-Banos M, John A, Petkov N, Berridge DM, Southern K, LLoyd K, et al. Using neural networks with routine health records to identify suicide risk: feasibility study. JMIR Ment Health. 2018;5(2):e10144. doi: 10.2196/10144 29934287 PMC6035342

[49] Edgcomb JB, Shaddox T, Hellemann G, Brooks JO 3rd. Predicting suicidal behavior and self-harm after general hospitalization of adults with serious mental illness. J Psychiatr Res. 2021;136:515–21. doi: 10.1016/j.jpsychires.2020.10.024 33218748 PMC8009812

[50] Edgcomb JB, Thiruvalluru R, Pathak J, Brooks JO 3rd. Machine learning to differentiate risk of suicide attempt and self-harm after general medical hospitalization of women with mental illness. Med Care. 2021;59:S58–64. doi: 10.1097/MLR.0000000000001467 33438884 PMC7810157

[51] Edgcomb JB, Tseng C-H, Pan M, Klomhaus A, Zima BT. Assessing detection of children with suicide-related emergencies: evaluation and development of computable phenotyping approaches. JMIR Ment Health. 2023;10:e47084. doi: 10.2196/47084 37477974 PMC10403798

[52] Fernandes AC, Dutta R, Velupillai S, Sanyal J, Stewart R, Chandran D. Identifying suicide ideation and suicidal attempts in a psychiatric clinical research database using natural language processing. Sci Rep. 2018;8(1):7426. doi: 10.1038/s41598-018-25773-2 29743531 PMC5943451

[53] Gholi Zadeh Kharrat F, Gagne C, Lesage A, Gariépy G, Pelletier J-F, Brousseau-Paradis C, et al. Explainable artificial intelligence models for predicting risk of suicide using health administrative data in Quebec. PLoS One. 2024;19(4):e0301117. doi: 10.1371/journal.pone.0301117 38568987 PMC10990247

[54] Gradus JL, Rosellini AJ, Horváth-Puhó E, Jiang T, Street AE, Galatzer-Levy I, et al. Predicting sex-specific nonfatal suicide attempt risk using machine learning and data from danish national registries. Am J Epidemiol. 2021;190(12):2517–27. doi: 10.1093/aje/kwab112 33877265 PMC8796814

[55] Gradus JL, Rosellini AJ, Horváth-Puhó E, Street AE, Galatzer-Levy I, Jiang T, et al. Prediction of sex-specific suicide risk using machine learning and single-payer health care registry data from Denmark. JAMA Psychiatry. 2020;77(1):25–34. doi: 10.1001/jamapsychiatry.2019.2905 31642880 PMC6813578

[56] Haroz EE, Rebman P, Goklish N, Garcia M, Suttle R, Maggio D, et al. Performance of machine learning suicide risk models in an American Indian population. JAMA Netw Open. 2024;7(10):e2439269. doi: 10.1001/jamanetworkopen.2024.39269 39401036 PMC11474420

[57] Jiang T, Nagy D, Rosellini AJ, Horváth-Puhó E, Keyes KM, Lash TL, et al. Prediction of suicide attempts among persons with depression: a population-based case cohort study. Am J Epidemiol. 2024;193(6):827–34. doi: 10.1093/aje/kwad237 38055633 PMC11466851

[58] Jiang T, Rosellini AJ, Horváth-Puhó E, Shiner B, Street AE, Lash TL, et al. Using machine learning to predict suicide in the 30 days after discharge from psychiatric hospital in Denmark. Br J Psychiatry. 2021;219(2):440–7. doi: 10.1192/bjp.2021.19 33653425 PMC8457342

[59] Kessler RC, Bauer MS, Bishop TM, Demler OV, Dobscha SK, Gildea SM, et al. Using administrative data to predict suicide after psychiatric hospitalization in the veterans health administration system. Front Psychiatry. 2020;11:390. doi: 10.3389/fpsyt.2020.00390 32435212 PMC7219514

[60] Martinez-Romo J, Araujo L, Reneses B. Guardian-BERT: early detection of self-injury and suicidal signs with language technologies in electronic health reports. Comput Biol Med. 2025;186:109701. doi: 10.1016/j.compbiomed.2025.109701 39967190

[61] Metzger M-H, Tvardik N, Gicquel Q, Bouvry C, Poulet E, Potinet-Pagliaroli V. Use of emergency department electronic medical records for automated epidemiological surveillance of suicide attempts: a French pilot study. Int J Methods Psychiatr Res. 2017;26(2):e1522. doi: 10.1002/mpr.1522 27634457 PMC6877202

[62] Nielsen SD, Christensen RHB, Madsen T, Karstoft K-I, Clemmensen L, Benros ME. Prediction models of suicide and non-fatal suicide attempt after discharge from a psychiatric inpatient stay: a machine learning approach on nationwide Danish registers. Acta Psychiatr Scand. 2023;148(6):525–37. doi: 10.1111/acps.13629 37961014

[63] O’Reilly LM, Fazel S, Rickert ME, Kuja-Halkola R, Cederlof M, Hellner C, et al. Evaluating machine learning for predicting youth suicidal behavior up to 1 year after contact with mental-health specialty care. Clin Psychol Sci. 2025;13(3):614–31. doi: 10.1177/21677026241301298 40771879 PMC12327383

[64] Obeid JS, Dahne J, Christensen S, Howard S, Crawford T, Frey LJ, et al. Identifying and predicting intentional self-harm in electronic health record clinical notes: deep learning approach. JMIR Med Inform. 2020;8(7):e17784. doi: 10.2196/17784 32729840 PMC7426805

[65] Penfold RB, Johnson E, Shortreed SM, Ziebell RA, Lynch FL, Clarke GN, et al. Predicting suicide attempts and suicide deaths among adolescents following outpatient visits. J Affect Disord. 2021;294:39–47. doi: 10.1016/j.jad.2021.06.057 34265670 PMC8820270

[66] Sanderson M, Bulloch AG, Wang J, Williams KG, Williamson T, Patten SB. Predicting death by suicide following an emergency department visit for parasuicide with administrative health care system data and machine learning. EClinicalMedicine. 2020;20:100281. doi: 10.1016/j.eclinm.2020.100281 32300738 PMC7152812

[67] Sanderson M, Bulloch AG, Wang J, Williamson T, Patten SB. Predicting death by suicide using administrative health care system data: can recurrent neural network, one-dimensional convolutional neural network, and gradient boosted trees models improve prediction performance?. J Affect Disord. 2020;264:107–14. doi: 10.1016/j.jad.2019.12.024 32056739

[68] Sheu Y-H, Simm J, Wang B, Lee H, Smoller JW. Continuous-time and dynamic suicide attempt risk prediction with neural ordinary differential equations. medRxiv. 2024:2024.02.25.24303343. doi: 10.1101/2024.02.25.24303343 40082653 PMC11906764

[69] Sheu Y-H, Sun J, Lee H, Castro VM, Barak-Corren Y, Song E, et al. An efficient landmark model for prediction of suicide attempts in multiple clinical settings. Psychiatry Res. 2023;323:115175. doi: 10.1016/j.psychres.2023.115175 37003169 PMC10267893

[70] Shortreed SM, Walker RL, Johnson E, Wellman R, Cruz M, Ziebell R, et al. Complex modeling with detailed temporal predictors does not improve health records-based suicide risk prediction. NPJ Digit Med. 2023;6(1):47. doi: 10.1038/s41746-023-00772-4 36959268 PMC10036475

[71] Simon GE, Johnson E, Lawrence JM, Rossom RC, Ahmedani B, Lynch FL, et al. Predicting suicide attempts and suicide deaths following outpatient visits using electronic health records. Am J Psychiatry. 2018;175(10):951–60. doi: 10.1176/appi.ajp.2018.17101167 29792051 PMC6167136

[72] Simon GE, Johnson E, Shortreed SM, Ziebell RA, Rossom RC, Ahmedani BK, et al. Predicting suicide death after emergency department visits with mental health or self-harm diagnoses. Gen Hosp Psychiatry. 2024;87:13–9. doi: 10.1016/j.genhosppsych.2024.01.009 38277798 PMC10939795

[73] Simon GE, Shortreed SM, Johnson E, Yaseen ZS, Stone M, Mosholder AD, et al. Predicting risk of suicidal behavior from insurance claims data vs. linked data from insurance claims and electronic health records. Pharmacoepidemiol Drug Saf. 2024;33(1):e5734. doi: 10.1002/pds.5734 38112287 PMC10843611

[74] Su C, Aseltine R, Doshi R, Chen K, Rogers SC, Wang F. Machine learning for suicide risk prediction in children and adolescents with electronic health records. Transl Psychiatry. 2020;10(1):413. doi: 10.1038/s41398-020-01100-0 33243979 PMC7693189

[75] Tsui FR, Shi L, Ruiz V, Ryan ND, Biernesser C, Iyengar S, et al. Natural language processing and machine learning of electronic health records for prediction of first-time suicide attempts. JAMIA Open. 2021;4(1):ooab011. doi: 10.1093/jamiaopen/ooab011 33758800 PMC7966858

[76] van Mens K, Elzinga E, Nielen M, Lokkerbol J, Poortvliet R, Donker G, et al. Applying machine learning on health record data from general practitioners to predict suicidality. Internet Interv. 2020;21:100337. doi: 10.1016/j.invent.2020.100337 32944503 PMC7481555

[77] Walsh CG, Ribeiro JD, Franklin JC. Predicting risk of suicide attempts over time through machine learning. Clin Psychol Sci. 2017;5(3):457–69. doi: 10.1177/2167702617691560

[78] Wang J, Qiu J, Zhu T, Zeng Y, Yang H, Shang Y, et al. Prediction of suicidal behaviors in the middle-aged population: machine learning analyses of UK Biobank. JMIR Public Health Surveill. 2023;9:e43419. doi: 10.2196/43419 36805366 PMC9989910

[79] Wilimitis D, Turer RW, Ripperger M, McCoy AB, Sperry SH, Fielstein EM, et al. Integration of face-to-face screening with real-time machine learning to predict risk of suicide among adults. JAMA Netw Open. 2022;5(5):e2212095. doi: 10.1001/jamanetworkopen.2022.12095 35560048 PMC9107032

[80] Xu W, Su C, Li Y, Rogers S, Wang F, Chen K, et al. Improving suicide risk prediction via targeted data fusion: proof of concept using medical claims data. J Am Med Inform Assoc. 2022;29(3):500–11. doi: 10.1093/jamia/ocab209 34850890 PMC8800522

[81] Xu Z, Zhang Q, Yip PSF. Predicting post-discharge self-harm incidents using disease comorbidity networks: a retrospective machine learning study. J Affect Disord. 2020;277:402–9. doi: 10.1016/j.jad.2020.08.044 32866798

[82] Yang Z, Mitra A, Hu W, Berlowitz D, Yu H. NLP-enriched social determinants of health improve prediction of suicide death among the Veterans. Res Sq. 2025:rs.3.rs-5067562. doi: 10.21203/rs.3.rs-5067562/v1 40235516 PMC11998781

[83] Zang C, Hou Y, Lyu D, Jin J, Sacco S, Chen K, et al. Accuracy and transportability of machine learning models for adolescent suicide prediction with longitudinal clinical records. Transl Psychiatry. 2024;14(1):316. doi: 10.1038/s41398-024-03034-3 39085206 PMC11291985

[84] Zheng L, Wang O, Hao S, Ye C, Liu M, Xia M, et al. Development of an early-warning system for high-risk patients for suicide attempt using deep learning and electronic health records. Transl Psychiatry. 2020;10(1):72. doi: 10.1038/s41398-020-0684-2 32080165 PMC7033212

[85] Deeks JJ, Altman DG. Diagnostic tests 4: likelihood ratios. BMJ. 2004;329(7458):168–9. doi: 10.1136/bmj.329.7458.168 15258077 PMC478236

[86] UK National Screening Committee. Criteria for a population screening programme; 2022. Available from: https://www.gov.uk/government/publications/evidence-review-criteria-national-screening-programmes/criteria-for-appraising-the-viability-effectiveness-and-appropriateness-of-a-screening-programme

[87] Dobrow MJ, Hagens V, Chafe R, Sullivan T, Rabeneck L. Consolidated principles for screening based on a systematic review and consensus process. CMAJ. 2018;190(14):E422–9. doi: 10.1503/cmaj.171154 29632037 PMC5893317

[88] Ross EL, Zuromski KL, Reis BY, Nock MK, Kessler RC, Smoller JW. Accuracy requirements for cost-effective suicide risk prediction among primary care patients in the US. JAMA Psychiatry. 2021;78(6):642–50. doi: 10.1001/jamapsychiatry.2021.0089 33729432 PMC7970389

[89] Belsher BE, Smolenski DJ, Pruitt LD, Bush NE, Beech EH, Workman DE, et al. Prediction models for suicide attempts and deaths: a systematic review and simulation. JAMA Psychiatry. 2019;76(6):642–51. doi: 10.1001/jamapsychiatry.2019.0174 30865249

[90] Hawton K, Lascelles K, Pitman A, Gilbert S, Silverman M. Assessment of suicide risk in mental health practice: shifting from prediction to therapeutic assessment, formulation, and risk management. Lancet Psychiatry. 2022;9(11):922–8. doi: 10.1016/S2215-0366(22)00232-2 35952701

[91] Witt KG, Hetrick SE, Rajaram G, Hazell P, Taylor Salisbury TL, Townsend E, et al. Psychosocial interventions for self-harm in adults. Cochrane Database Syst Rev. 2021;4(4):CD013668. doi: 10.1002/14651858.CD013668.pub2 33884617 PMC8094743

[92] Milner AJ, Carter G, Pirkis J, Robinson J, Spittal MJ. Letters, green cards, telephone calls and postcards: systematic and meta-analytic review of brief contact interventions for reducing self-harm, suicide attempts and suicide. Br J Psychiatry. 2015;206(3):184–90. doi: 10.1192/bjp.bp.114.147819 25733570

[93] Nuij C, van Ballegooijen W, de Beurs D, Juniar D, Erlangsen A, Portzky G, et al. Safety planning-type interventions for suicide prevention: meta-analysis. Br J Psychiatry. 2021;219(2):419–26. doi: 10.1192/bjp.2021.50 35048835

[94] Cooper J, Steeg S, Bennewith O, Lowe M, Gunnell D, House A, et al. Are hospital services for self-harm getting better? An observational study examining management, service provision and temporal trends in England. BMJ Open. 2013;3(11):e003444. doi: 10.1136/bmjopen-2013-003444 24253029 PMC3840333

[95] Graney J, Hunt IM, Quinlivan L, Rodway C, Turnbull P, Gianatsi M, et al. Suicide risk assessment in UK mental health services: a national mixed-methods study. Lancet Psychiatry. 2020;7(12):1046–53. doi: 10.1016/S2215-0366(20)30381-3 33189221

[96] Mulder R, Newton-Howes G, Coid JW. The futility of risk prediction in psychiatry. Br J Psychiatry. 2016;209(4):271–2. doi: 10.1192/bjp.bp.116.184960 27698212

[97] Royal College of Psychiatrists. Self-harm, suicide and risk: helping people who self-harm: final report of a working group (College Report CR158). Royal College of Psychiatrists; 2010.

[98] Blasiak A, Khong J, Kee T. CURATE.AI: optimizing personalized medicine with artificial intelligence. SLAS Technol. 2020;25(2):95–105. doi: 10.1177/2472630319890316 31771394

