# Supplementary material for: Machine learning algorithms and their predictive accuracy for suicide and self-harm: Systematic review and meta-analysis
Source: PLoS Med. 2025 Sep 11;22(9):e1004581. doi: 10.1371/journal.pmed.1004581 (PMC12425223; doi:10.1371/journal.pmed.1004581)
Supplement: S1 Table — (S1_Table.DOCX) [file pmed.1004581.s002.docx]

S1 Table: TRIPOD checklist adherence for 53 included studies

| **Item** | **Yes** | **No** | **Not relevant** |
| --- | --- | --- | --- |
| Identify the study as developing and/or validating a multivariable prediction model, the target population, and the outcome to be predicted | 36 | 17 | 0 |
| Provide a summary of objectives, study design, setting, participants, sample size, predictors, outcome, statistical analysis, results, and conclusions | 34 | 19 | 0 |
| Explain the medical context (including whether diagnostic or prognostic) and rationale for developing or validating the multivariable prediction model, including references to existing models | 50 | 3 | 0 |
| Specify the objectives, including whether the study describes the development or validation of the model, or both | 48 | 5 | 0 |
| Describe the study design or source of data (for example, randomised trial, cohort, or registry data), separately for the development and validation data sets, if applicable | 48 | 5 | 0 |
| Specify the key study dates, including start of accrual; end of accrual; and, if applicable, end of follow-up | 48 | 5 | 0 |
| Specify key elements of the study setting (for example, primary care, secondary care, general population) including number and location of centres | 42 | 11 | 0 |
| Describe eligibility criteria for participants | 48 | 5 | 0 |
| Give details of treatments received, if relevant* | 2 | 2 | 49 |
| Clearly define the outcome that is predicted by the prediction model, including how and when assessed | 44 | 9 | 0 |
| Report any actions to blind assessment of the outcome to be predicted* | 1 | 15 | 37 |
| Clearly define all predictors used in developing the multivariable prediction model, including how and when they were measured | 38 | 14 | 1 |
| Report any actions to blind assessment of predictors for the outcome and other predictors* | 1 | 15 | 37 |
| Explain how the study size was arrived at. | 11 | 42 | 0 |
| Describe how missing data were handled (for example, complete-case analysis, single imputation, multiple imputation) with details of any imputation method | 19 | 34 | 0 |
| Describe how predictors were handled in the analyses | 44 | 9 | 0 |
| Specify type of model, all model-building procedures (including any predictor selection), and method for internal validation | 49 | 4 | 0 |
| Specify all measures used to assess model performance and, if relevant, to compare multiple models | 51 | 2 | 0 |
| Provide details on how risk groups were created, if done | 11 | 40 | 2 |
| Describe the flow of participants through the study, including the number of participants with and without the outcome and, if applicable, a summary of the follow-up time. | 44 | 8 | 1 |
| Describe the characteristics of the participants (basic demographics, clinical features, available predictors), including the number of participants with missing data for predictors and outcome | 40 | 13 | 0 |
| Specify the number of participants and outcome events in each analysis | 47 | 6 | 0 |
| If done, report the unadjusted association between each candidate predictor and outcome | 10 | 41 | 2 |
| Present the full prediction model to allow predictions for individuals (that is, all regression coefficients, and model intercept or baseline survival at a given time point) | 25 | 28 | 0 |
| Explain how to use the prediction model | 23 | 28 | 2 |
| Report performance measures (with CIs) for the prediction model | 43 | 10 | 0 |
| Discuss any limitations of the study (such as nonrepresentative sample, few events per predictor, missing data) | 47 | 6 | 0 |
| Give an overall interpretation of the results, considering objectives, limitations, results from similar studies, and other relevant evidence | 50 | 3 | 0 |
| Discuss the potential clinical use of the model and implications for future research | 48 | 5 | 0 |
| Provide information about the availability of supplementary resources, such as study protocol, Web calculator, and data sets | 33 | 20 | 0 |
| Give the source of funding and the role of the funders for the present study | 37 | 16 | 0 |

Note: * items removed from the calculation of adherence for each study
