# Supplementary material for: Machine learning algorithms and their predictive accuracy for suicide and self-harm: Systematic review and meta-analysis
Source: PLoS Med. 2025 Sep 11;22(9):e1004581. doi: 10.1371/journal.pmed.1004581 (PMC12425223; doi:10.1371/journal.pmed.1004581)
Supplement: S2 Table — (S2_Table.DOCX) [file pmed.1004581.s003.docx]

S2 Table: Risk of bias ratings for each study

| **Study** | **Patient selection** | **Index test** | **Reference standard** | **Flow and timing** | **Overall risk of bias** |
| --- | --- | --- | --- | --- | --- |
| Amini 2016 | Unclear | Unclear | Unclear | Unclear | Unclear |
| Arora 2023 | Low | Unclear | Unclear | Unclear | Unclear |
| Barak-Corren 2017 | Low | Unclear | Unclear | Unclear | Unclear |
| Barak-Corren 2020 | Unclear | Unclear | Low | Low | Unclear |
| Barak-Corren 2023 | Low | Unclear | Low | Unclear | Unclear |
| Ben-Ari 2015 | High | Unclear | High | Unclear | High |
| Bentley 2025 | Unclear | Unclear | High | Low | High |
| Bittar 2019 | High | Unclear | Unclear | Low | High |
| Cansel 2023 | High | Unclear | Low | Unclear | High |
| Carson 2024 | Unclear | Unclear | Unclear | Low | Unclear |
| Chen 2020 | Low | Unclear | Low | Low | Unclear |
| Chen 2024 | High | Unclear | Unclear | High | High |
| Cho 2020 | High | Unclear | Unclear | High | High |
| Cho 2021 | High | Unclear | Unclear | High | High |
| Coley 2021 | Low | Unclear | Unclear | Unclear | Unclear |
| Coley 2023 | Low | Unclear | Unclear | Unclear | Unclear |
| DelPozo-Banos 2018 | High | Unclear | Low | Low | High |
| Edgcomb 2021a | High | Unclear | Unclear | Unclear | High |
| Edgcomb 2021b | High | Unclear | Unclear | Unclear | High |
| Edgcomb 2023 | High | High | Unclear | Unclear | High |
| Fernandes 2018 | High | Unclear | Unclear | Unclear | High |
| Gholi Zadeh Kharrat 2024 | High | Unclear | Low | High | High |
| Gradus 2020 | Low | Unclear | Unclear | Unclear | Unclear |
| Gradus 2021 | Low | Unclear | Unclear | Unclear | Unclear |
| Haroz 2024 | Low | Unclear | Unclear | Low | Unclear |
| Jiang 2021 | Low | Unclear | Unclear | Unclear | Unclear |
| Jiang 2024 | Low | Unclear | Low | Unclear | Unclear |
| Kessler 2020 | High | Unclear | Low | Low | High |
| Martinez-Romo 2025 | High | Unclear | High | Unclear | High |
| Metzger 2017 | High | Unclear | Unclear | Unclear | High |
| Nielsen 2023 | Low | High | Unclear | Unclear | High |
| O'Reilly 2024 | Low | Unclear | Unclear | Low | Unclear |
| Obeid 2020 | High | Unclear | High | Unclear | High |
| Penfold 2021 | Low | Unclear | Unclear | Low | Unclear |
| Sanderson 2020a | High | Unclear | Low | Unclear | High |
| Sanderson 2020b | High | Unclear | Low | Unclear | High |
| Sheu 2023 | High | Unclear | Unclear | Low | High |
| Sheu 2024 | Unclear | Unclear | Low | Unclear | Unclear |
| Shortreed 2023 | Unclear | Unclear | Low | Low | Unclear |
| Simon 2018 | Low | Low | Low | Low | Low |
| Simon 2024a | Unclear | Low | Unclear | Low | Unclear |
| Simon 2024b | Unclear | Unclear | Low | Low | Unclear |
| Su 2020 | Low | Low | Low | Low | Low |
| Tsui 2021 | High | Unclear | Unclear | Unclear | High |
| Van Mens 2020 | High | High | High | Low | High |
| Walsh 2017 | High | Low | Unclear | Unclear | High |
| Wang 2023 | High | Unclear | Low | Unclear | High |
| Wilimitis 2022 | Unclear | Unclear | Unclear | Unclear | Unclear |
| Xu 2020 | High | Unclear | Unclear | Unclear | High |
| Xu 2022 | High | Unclear | Unclear | Unclear | High |
| Yang 2025 | Low | Low | Low | Low | Low |
| Zang 2024 | Unclear | Unclear | Unclear | Unclear | Unclear |
| Zheng 2020 | Unclear | Low | Unclear | Low | Unclear |
