# Supplementary material for: Machine learning algorithms and their predictive accuracy for suicide and self-harm: Systematic review and meta-analysis
Source: PLoS Med. 2025 Sep 11;22(9):e1004581. doi: 10.1371/journal.pmed.1004581 (PMC12425223; doi:10.1371/journal.pmed.1004581)
Supplement: S1 Fig — (DOCX) [file pmed.1004581.s004.docx]

S1 Fig: sROC curves of machine learning instruments to predict suicide, self-harm, suicide/self-harm
